# Multicenter experience of hybrid transcatheter pulmonary valve implantation in patients with a large right ventricular outflow tract

**DOI:** 10.3389/fcvm.2026.1815136

**Published:** 2026-10-20

**Authors:** Jun Dong, Junwu Su, Aijun Liu, Shusheng Wen, Taibing Fan, Qi An, Bing Jia, Ming Ye, Gangcheng Zhang, Yongqiang Jin, Yaxiong Li, Cheng Zhou, Baoshi Zheng, Jing Xu

**Affiliations:** 1 Department of Pediatric Cardiac Center, Beijing Anzhen Hospital, Capital Medical University, Beijing, China; 2 Department of Cardiovascular Surgery, Guangdong Provincial People’s Hospital, Guangzhou, China; 3 Department of Cardiovascular Surgery, Fuwai Central China Cardiovascular Hospital, Zhengzhou, China; 4 Department of Cardiovascular Surgery, West China Hospital, Sichuan University, Chengdu, China; 5 Department of Cardiovascular Surgery, Children’s Hospital of Fudan University, Shanghai, China; 6 Department of Cardiovascular Surgery, Zhongnan Hospital of Wuhan University, Wuhan, China; 7 Department of Cardiovascular Surgery, Beijng Huaxin Hospital First Hospital of Tsinghua University, Beijing, China; 8 Department of Cardiovascular Surgery, Kunming shi Yan'an Hospital, Kunming, China; 9 Department of Cardiovascular Surgery, Union Hospital, Tongji Medical College, Huazhong University of Science and Technology, Wuhan, China; 10 Department of Cardiovascular Surgery, The First Affiliated Hospital of Guangxi Medical University, Nanning, China; 11 Department of Cardiovascular Surgery, The First Affiliated Hospital of Zhengzhou University, Zhengzhou, China

**Keywords:** hybrid transcatheter pulmonary valve implantation, native right ventricular outflow tract reconstruction, pulmonary regurgitation, right ventricular outflow tract morphology, transcatheter pulmonary valve implantation

## Abstract

**Background:**

Management of pulmonary regurgitation (PR) in patients with a large and dysplastic native right ventricular outflow tract (RVOT) remains a significant challenge for percutaneous intervention. Given the limitations of percutaneous intervention in patients with a large and morphologically complex RVOT, this study aimed to evaluate the safety and efficacy of a hybrid transcatheter pulmonary valve implantation (TPVI) strategy using a self-expanding valve in this patient population.

**Methods:**

Between June 2021 and December 2023, 102 patients with moderate-to-severe PR and dilated RVOT from 11 centers underwent hybrid TPVI with the Salus transcatheter pulmonary valve (TPV). Procedural strategies, including surgical access and concomitant procedures, were tailored based on comprehensive preoperative assessment.

**Results:**

The mean age was 22.37 ± 11.15 years (range, 9–66), and 56.9% were male. The most common RVOT morphologies were pyramidal (37.2%) and tubular (30.4%). All procedures were successful. Twenty cases required cardiopulmonary bypass support. There were no intraoperative deaths. One in-hospital death occurred on postoperative day 12 in a high-risk patient with severe preoperative right ventricular dysfunction. All patients completed at least 1 year of follow-up, with a median follow-up duration of 1 year. During this period, significant improvements in right ventricular volumes (RVEDVI and RVESVI) and QRS duration were observed, whereas RVEF did not change significantly. There were no instances of coronary artery compression, valve embolization, or stent fracture. One suspected case of endocarditis was reported.

**Conclusions:**

In patients with a large and morphologically complex RVOT, hybrid TPVI with the Salus TPV is a safe and effective therapeutic strategy, associated with high procedural success and encouraging short-term outcomes. A patient-specific approach, guided by meticulous preoperative imaging to determine the optimal access and the need for concomitant procedures, is key to minimizing complications. This hybrid technique offers a valuable alternative for patients who are not candidates for percutaneous intervention.

## 1 Introduction

Right ventricular outflow tract (RVOT) dysplasia associated with congenital heart disease such as tetralogy of Fallot (TOF), pulmonary stenosis, pulmonary atresia with intact ventricular septum, and double outlet right ventricle require native right ventricular outflow reconstruction (NRVOTR) during surgical repair. Depending on patients' RVOT pathology, reconstruction is achieved by pulmonary valvotomy, infundibulectomy, patch repair or transannular patch (1). Pulmonary regurgitation (PR) is the most common late postoperative complication after NRVOTR (2). In recent years, several clinical trials have been conducted worldwide on self-expanding percutaneous TPVI in patients after NRVOTR, and encouraging follow-up results have been achieved. However, due to the constraints of large RVOT and irregular morphology, the problems of low patient screening acceptance rate, possible intraoperative valve displacement and embolization, oversized valve diameter, and tricuspid valve injury cannot be ignored in percutaneous TPVI (3–9).

Several recent studies have explored hybrid approaches with balloon-expandable pulmonary valves, reporting promising outcomes; however, evidence remains limited by small cohort sizes (10–16). Therefore, we summarize the experience of hybrid transcatheter pulmonary valve implantation (TPVI) in a Chinese multicenter study to describe in detail the demographic information, morphological characteristics of the RVOT, surgical protocol, and follow-up results.

## 2 Methods

### 2.1 Patients

This multicenter, retrospective observational study included 102 consecutive patients who underwent hybrid TPVI with the Salus valve between June 2021 and December 2023 at 11 Chinese centers as routine clinical care. The procedure indication was determined by each center's multidisciplinary heart team, independent of any research protocol. We queried each center's surgical and imaging databases to identify all hybrid TPVI recipients. The following eligibility criteria were then applied retrospectively to assemble a homogeneous analysis cohort. These criteria were not used for prospective patient selection.

The inclusion criteria were: (1) moderate-to-severe PR diagnosed by transthoracic echocardiography (which was graded in accordance with the American Society of Echocardiography recommendations for native valves); (2) patients had symptoms associated with RVOT dysfunction after other diseases with identical symptoms had been excluded (dyspnea, syncope, palpitations, reduced tolerance to exercise); (3) patients may be asymptomatic but present with one of the following conditions: moderate-to-severe functional tricuspid regurgitation (TR) upon transthoracic echocardiography; right ventricular end-diastolic volume index (RVEDVI) ≥160 mL/m^2^ or right ventricular end-systolic volume index (RVESVI) ≥80 mL/m^2^ or right ventricular ejection fraction (RVEF) <45% as measured by CMRI; QRS width ≥160 ms.

The exclusion criteria were: (1) presence of an RVOT with conduit from the right ventricle (RV) to the pulmonary artery (PA) (2) lesions due to residual RVOT obstruction necessitating surgical intervention; (3) anatomic inadequacies [e.g., high risk of coronary artery compression (CAC) diagnosed by angiography].

This retrospective study was conducted in accordance with the Declaration of Helsinki. The study protocol was part of a previously approved prospective trial (IRB 2020–052I, Beijing Anzhen Hospital, February 23, 2021), the scope of which included secondary analyses of collected data. The ethics committee confirmed that this secondary analysis was covered by the original approval and granted a waiver of informed consent due to its retrospective design, minimal risk, and the use of de-identified data.

### 2.2 RVOT morphology

RVOT morphology was evaluated to calculate its anchor diameter (a key factor for selection of the optimal valve size and release position). Gated spiral CTA can provide accurate RVOT morphology parameters (17). The anatomy of the RVOT (length, diameter) at baseline was recorded. Measurements of the RVOT were performed on double oblique reconstructed images at five levels: the proximal end of the funnel sleeve, the fusion part, the midpoint between these two, the proximal pulmonary trunk (PT), and the midpoint between the PT and the fusion part. The RVOT diameter during the full cardiac cycle was recorded by ECG-gating CTA. According to the systolic sequence, the RVOT diameter was calculated by the circumference evaluation of the inner diameter. Three-dimensional CTA images of 102 patients were quantitatively classified into distinct RVOT shapes. We employed a classification method modified from that of Schievano and colleagues (Table 1) (18). The average diameter ®y along the RVOT length was determined. The difference between diameters was calculated and expressed as a percentage of the average RVOT diameter. Coronary artery anatom was evaluated in all patients using ECG gate CTA. The spatial relationship between the intended landing zone and adjacent coronary arteries was carefully assessed. An interarterial distance of less than 5 mm was considered a potential risk for CAC; such patients underwent selective coronary angiography with concurrent test balloon inflation at the intended landing zone diameter.

**Table 1:** Classification of right ventricular outflow tract morphology based on computed tomography measurements.

Morphology type	Descriptive name	Definition (based on percentage changes in diameter)
Type I	Pyramidal	Δy1 > −10% and Δy2 > −10% and (Δy1 + Δy2 > 10% or Δy1 > 10% or Δy2 > 10%)
Type II	Tubular	−10% ≤ Δy1 ≤ 10% and −10% ≤ Δy2 ≤ 10% and −10% ≤ Δy1 + Δy2 ≤ 10%
Type III	Funnel	Δy1 < 10% and Δy2 < 10% and (Δy1 + Δy2 < −10% or Δy1 < −10% or Δy2 < −10%)
Type IV	Lantern	Δy1 > 10% and Δy2 < −10%
Type V	Hourglass	Δy1 < −10% and Δy2 > 10%

Definitions of abbreviations and measurements: yA, diameter at the proximal part of the pulmonary trunk; yB, diameter at the mid-segment; yC, diameter at the fusion part (transition from infundibulum to main pulmonary artery); ȳ, average diameter along the measured RVOT length; Δy1 = (yB–yA)/ȳ × 100 (percentage change from proximal to mid); Δy2 = (yC–yB)/ȳ × 100 (percentage change from mid to fusion).

### 2.3 Salus transcatheter pulmonary valve (TPV)

In this study, TPVI was performed using the Salus TPV (Balance Medical Technology Co. Ltd., Beijing, China) and its corresponding delivery system (14–16). The Salus TPV is created by mounting three bovine pericardial leaflets on a self-expandable nitinol hourglass-shaped stent. The valve is available in inflow/outflow diameters from 22 to 36 mm (2 mm increments) and mid-section (leaflet attachment) diameters from 18 to 32 mm (2 mm increments). Total valve length ranges from 31 to 43 mm depending on the size. The delivery system requires a 22–24 Fr sheath.

The valve size shown in Table 3 refers to the mid-section (leaflet attachment) diameter, which ranges from 18 to 32 mm. The corresponding inflow/outflow diameters range from 22 to 36 mm. Therefore, a 32 mm valve in Table 3 indicates a mid-section diameter of 32 mm, not a native RVOT diameter of 32 mm.

The valve is indicated for a native RVOT landing zone diameter of 18–30 mm. For patients with a larger RVOT (≥30 mm where oversizing is required), intraoperative MPA constriction is performed to reduce the landing zone diameter to the appropriate range (see Procedural details).

### 2.4 Procedural details

Procedures were carried out under general anesthesia and endotracheal intubation in a hybrid operating theatre. Cardiopulmonary bypass (CPB) equipment was also present on standby. Hybrid surgical approaches included sternotomy, partial inferior sternotomy, left anterior thoracotomy and subxiphoid approach. The selection of the hybrid surgical approach was mainly based on the RVOT morphology and concurrent malformation requiring surgery (Figure 1). According to preoperative ventricular systolic CTA, the middle part of the main pulmonary artery (MPA) was selected for valve sizing with the diameter calculated by the circumference (Figure 2). The Salus TPV was released under the guidance of fluoroscopy (Figures 3, 4). For patients with a high risk of intraoperative paravalvular leakage (PVL) or dilated RVOT requiring downsizing (inner diameter ≥30 mm), anatomical dissection of the RVOT was first performed, followed by intraoperative constriction of the MPA at the mid-level intended as the landing zone for the Salus TPV using a constriction band of Gore-Tex patch with a width of 1 cm and length of 10 cm. Since the cylinder section of the Salus valve is approximately 15 mm in length, MPA constriction band with a width of 1 cm provides a safe and adequate valve landing zone. The length of 10 cm is chosen to allow for a 5 mm suture margin on each end, enabling the constriction band to form a circular landing zone with a diameter of approximately 30 mm. Following concomitant constriction and TPVI, the stent, constriction band, and the MPA wall at the landing zone were further secured together using continuous sutures of 4-0 polypropylene to prevent band migration and subsequent valve displacement. The sheath puncture site was selected on the bare area of the RV surface for intraoperative angiography and valve deployment. Depending on the operator's preference, some centers might also choose to perform angiography via the femoral vein. After valve deployment, the stability of the valve anchoring position, appropriate valve height, good coaxial alignment, and patency of blood flow in the left and right PA branches were assessed. Simultaneously, transesophageal echocardiography was performed to evaluate valve function and detect any PVL. If PVL was present, concurrent external plication of the MPA is performed without CPB. Based on the location of the leak identified by transesophageal echocardiography, a 4-0 polypropylene suture buttressed with a Dacron strip was used to suture the MPA wall directly onto the underlying Salus valve stent. This maneuver was performed during ventricular diastole to avoid needle injury to the pulmonary valve leaflets, which are open in systole. If valve migration affects tricuspid valve function, causes frequent ventricular tachycardia, or results in poor coaxiality due to rotation or inversion, the valve was explanted via MPA incision under CPB and then sutured for fixation.

**Figure 1:**
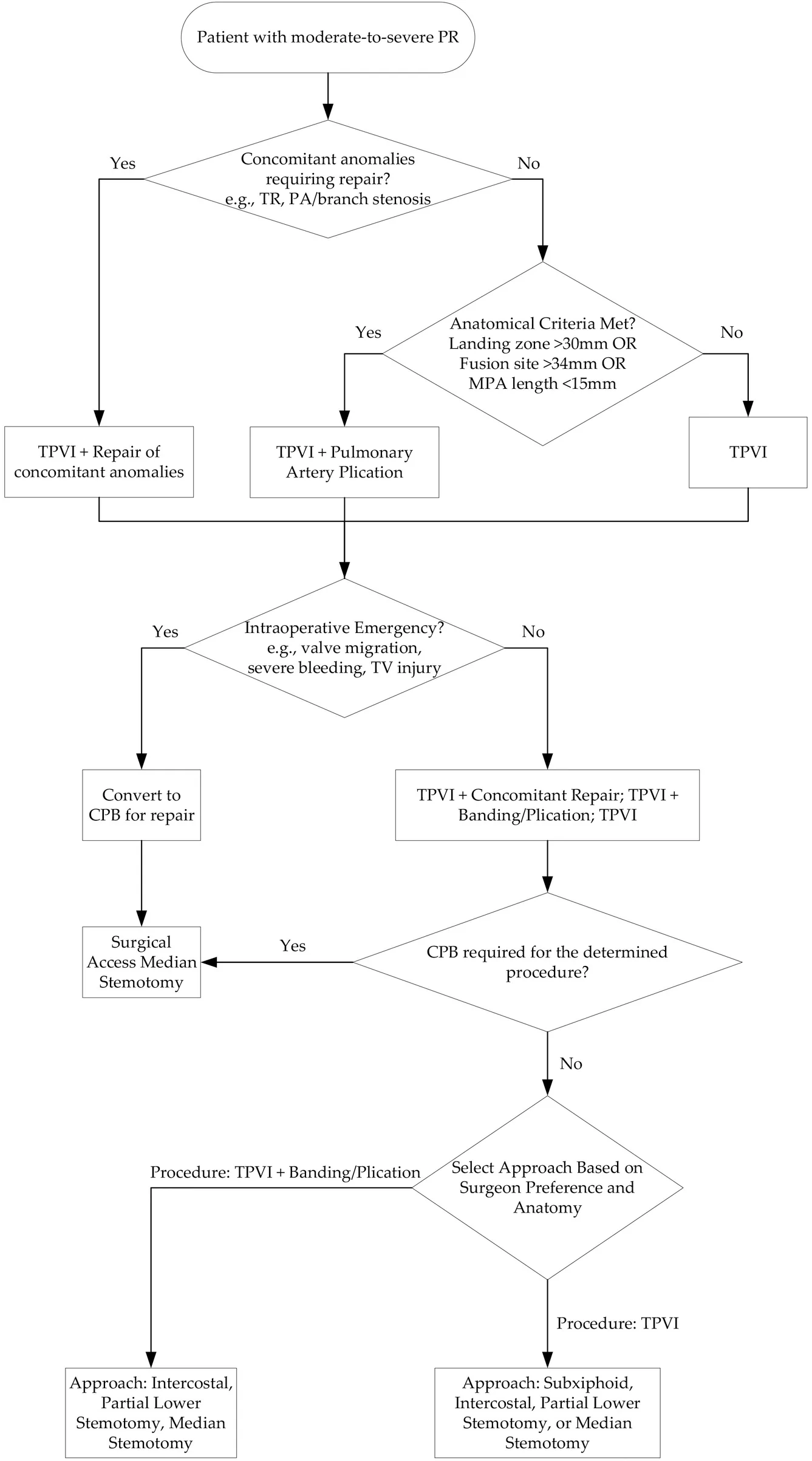
Flowchart depicting the decision-making process for operative strategy and approach in hybrid TPVI.

**Figure 2:**
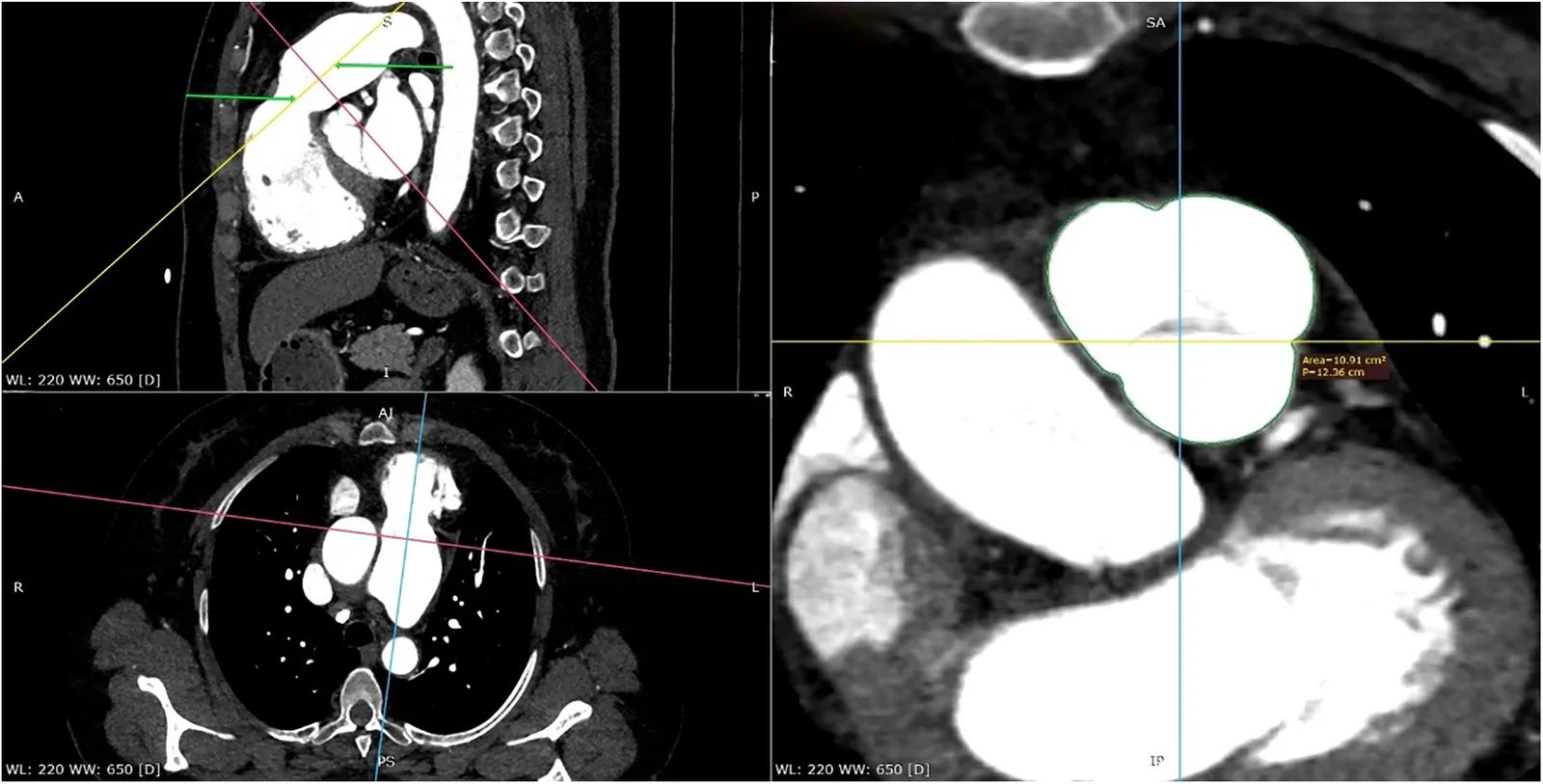
The diameter is calculated based on the circumference on the three-dimensional CTA images.

**Figure 3:**
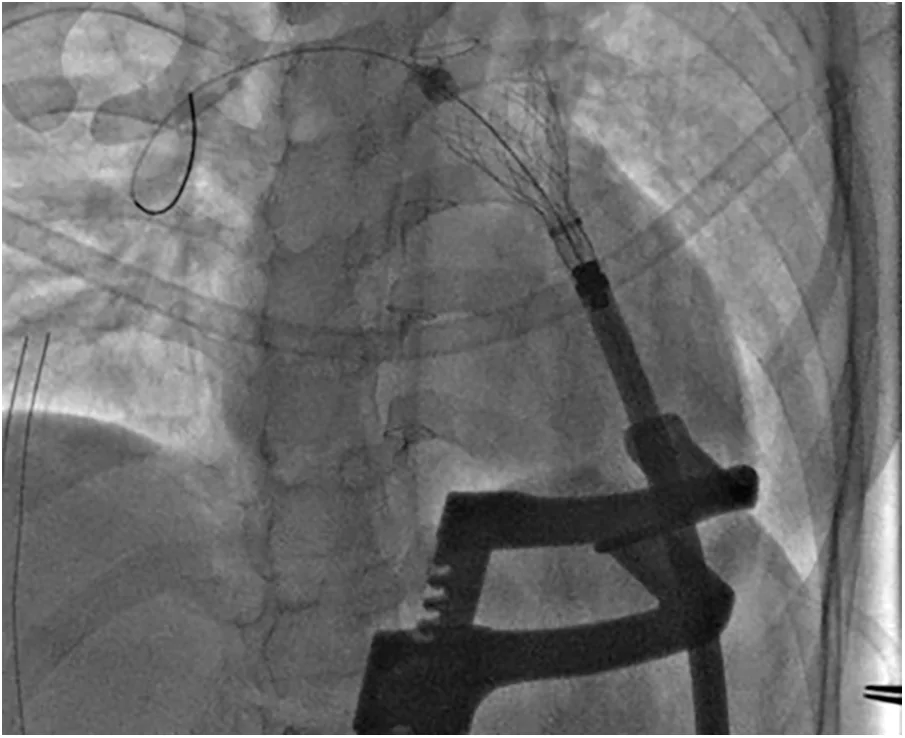
Intraoperative angiography showing deployment of the Salus TPV.

**Figure 4:**
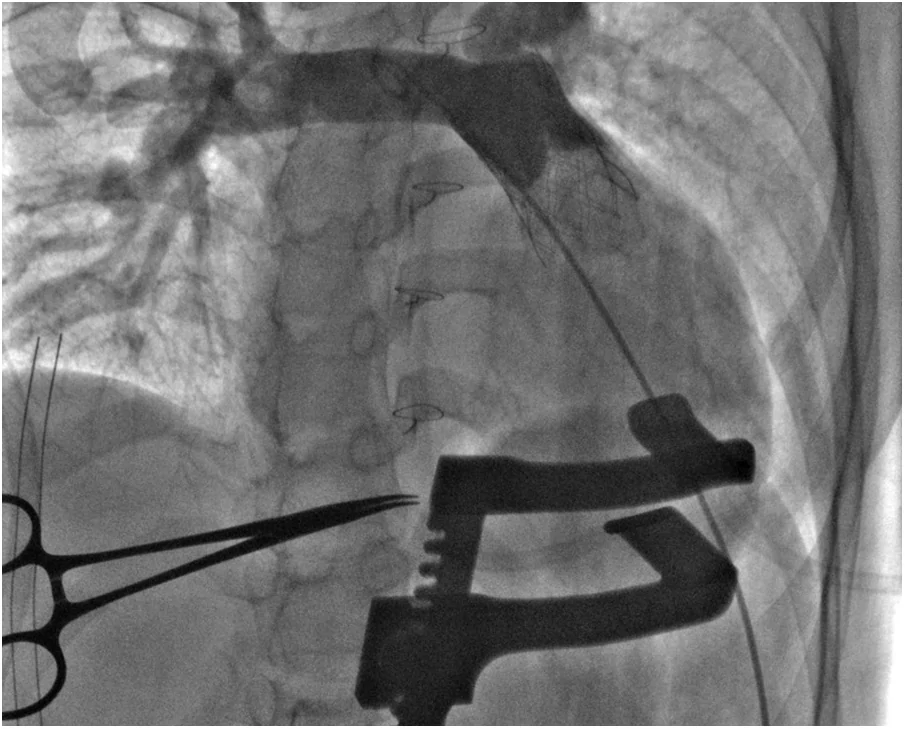
Post-deployment angiography confirming valve position and assessing paravalvular leakage.

### 2.5 Study design and clinical data collection

This was a multicenter, retrospective observational study (consecutive case series). Data were collected retrospectively via chart review from each institution's electronic medical records, operative notes, echocardiography reports, CTA and CMRI reports, discharge summaries, and outpatient follow-up records. A standardized data extraction form was used across all centers. No prospective registry was used for this analysis.

All participating centers are high-volume referral hospitals for adult congenital heart disease. The hybrid TPVI procedures were performed by dedicated multidisciplinary heart teams consisting of experienced congenital cardiac surgeons and interventional cardiologists. Prior to the study initiation, the procedural protocol and valve deployment technique were standardized across centers through dedicated training sessions.

At baseline, physical examination was undertaken and medical history documented. We also carried out routine hematology, analyses of blood biochemistry, coagulation function, urinalysis, and measured the level of C-reactive protein. Twelve-lead ECG, chest radiography, transthoracic echocardiography were also carried out.

All imaging studies, including CTA, CMRI, and transthoracic echocardiography, were assessed locally at each participating center by experienced congenital cardiac radiologists or cardiologists. No central core laboratory was used for this retrospective analysis. Inter-observer variability in RVOT measurements and echocardiographic assessments was not systematically evaluated.

### 2.6 Postoperative management

All patients returned to the cardiac intensive care unit that day for post-procedural monitoring. Prophylactic heparin at 15 units/kg/h was administered. Warfarin was commenced as soon as oral feeding was established and continued for 6 months with the Salus TPV *in situ*. INR was maintained between 1.8 and 2.5, with dose adjustments made based on regular monitoring. Postoperative follow-up was at 1, 3, 6, and 12 months, and then annually to assess valve function and potential postoperative complications.

### 2.7 Statistical analysis

Continuous variables are expressed as mean ± standard deviation or median (range) as appropriate. Categorical variables are presented as counts and percentages. Normality of continuous variables was assessed using the Shapiro–Wilk test. Normally distributed paired data were compared using paired *t*-tests, and non-normally distributed paired data were compared using Wilcoxon signed-rank tests. Inter-group comparisons were performed using ANOVA or Kruskal–Wallis tests for continuous variables, and Chi-square or Fisher's exact tests for categorical variables, as appropriate. Correlations were assessed using Pearson's correlation coefficient. As this was an exploratory retrospective study with multiple subgroup analyses, no adjustment for multiple comparisons was applied, and no *a priori* power calculation was performed. *P* values should therefore be interpreted as hypothesis-generating, and comparisons across RVOT morphology types may be underpowered to detect small but clinically relevant differences. A two-sided *P* value <0.05 was considered statistically significant. All analyses were performed using SPSS 24.0 software.

## 3 Results

### 3.1 Patient population

Demographic, clinical, and medical history are summarized in Supplementary Table S1. A total of 102 patients with moderate-to-severe PR with RVOT dilation underwent hybrid TPVI (Supplementary Table S5). The mean age was 22.37 ± 11.15 (range, 9–66) years, and 56.9% of patients were male. Among them, 99 patients had undergone one or two previous cardiac reoperations before the current hybrid TPVI. The mean time since the most recent operation was 15.22 ± 5.14 years.

### 3.2 RVOT morphology

The morphological characteristics of the RVOT are summarized in Table 2 and Figure 5. Based on the quantitative classification, the pyramidal shape (Type I) was the most prevalent morphology (37.2%), followed by the tubular shape (Type II, 30.4%). The mean length of the RVOT was 36.80 ± 7.43 mm, and the mean length of the PT was 26.40 ± 6.83 mm. A comparative analysis of pre- and post-operative parameters across the different RVOT morphology types revealed no statistically significant differences in functional outcomes, although expected differences in anatomical dimensions (infundibulum and distal RVOT diameters) were observed. The preoperative data and postoperative data of different RVOT morphology, except for the inner diameter, were not statistically significant (Supplementary Table S2).

**Table 2:** Morphological characteristics of the RVOT (*N* = 102).

Parameter	Value
RVOT length (mm), mean ± SD	36.80 ± 7.43
PT length (mm), mean ± SD	26.40 ± 6.83
Morphology type, *n* (%)	
Type I (pyramidal)	38 (37.2%)
Type II (tubular)	31 (30.4%)
Type III (funnel)	16 (15.7%)
Type IV (lantern)	6 (5.9%)
Type V (hourglass)	11 (10.8%)

RVOT, right ventricular outflow tract; SD, standard deviation; RVOT, length = proximal end of funnel sleeve to fusion; PT, pulmonary trunk; PT, length = proximal end of PT to fusion.

**Figure 5:**
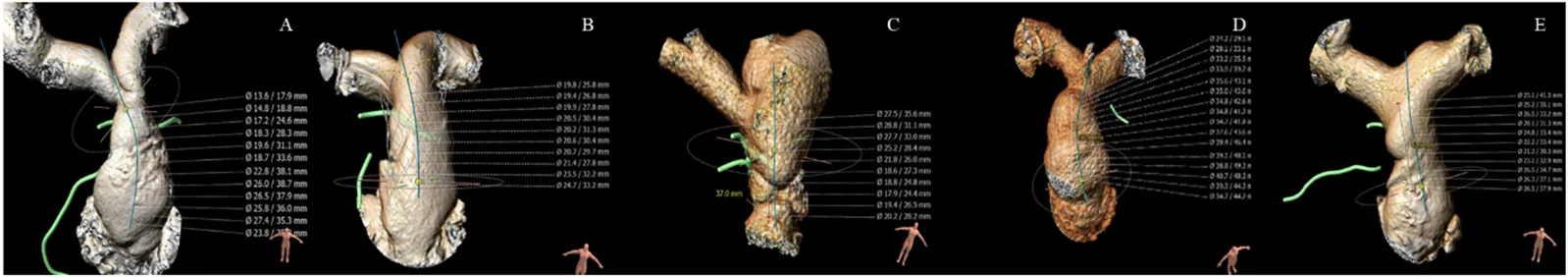
RVOT morphology distribution. **(A)** Pyramidal (proximally wide, distally narrow); **(B)** tubular (uniform diameter); **(C)** funnel (proximally narrow, distally wide); **(D)** lantern (widest centrally); **(E)** hourglass (narrowed centrally).

MPA constriction was performed in 24 patients (23.5%). The frequency of constriction across morphology types was: Type I, 10/38 (26.3%); Type II, 7/31 (22.6%); Type III, 3/16 (18.8%); Type IV, 1/6 (16.7%); Type V, 3/11 (27.3%). There was no statistically significant association between morphology type and the need for constriction (*p* = 0.89, Fisher's exact test), indicating that the decision was based primarily on the absolute inner diameter of the landing zone rather than the shape classification.

### 3.3 Correlations between RVOT anatomy and ventricular remodeling

To assess the pathophysiological link between RVOT anatomy and the degree of chronic volume overload, correlation analysis was performed. The proximal RVOT diameter (P-RVOT) was significantly correlated with greater pre-operative RVEDVI (*r* = 0.388, *p* < 0.01) and RVESVI (*r* = 0.379, *p* < 0.01). The mid-RVOT diameter (M-RVOT) was also significantly correlated with pre-operative RVEDVI (*r* = 0.314, *p* < 0.01). Figure 6 provides a comprehensive overview of the correlations among all morphological and functional parameters.

**Figure 6:**
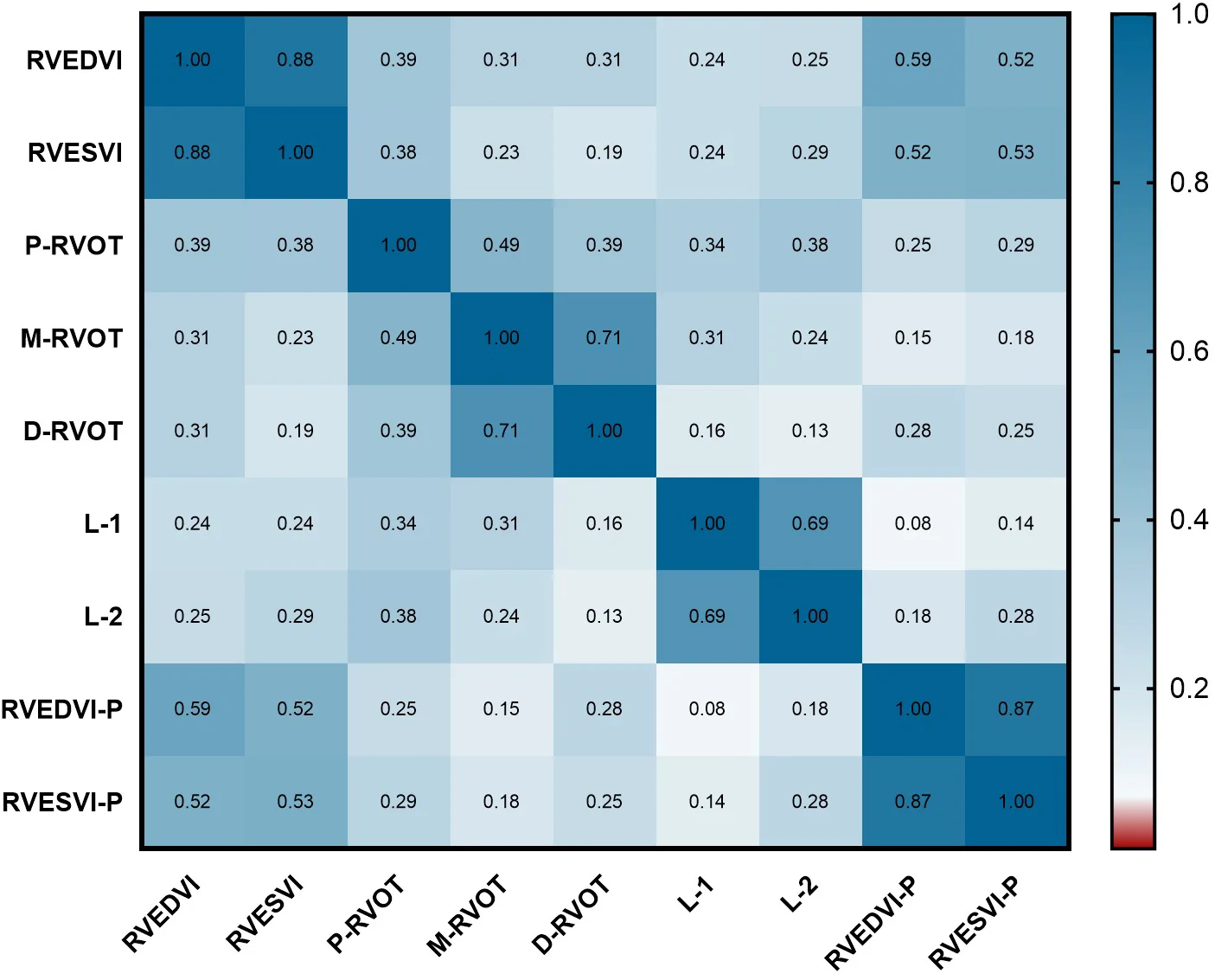
Correlation analysis of clinical and morphological parameters. Hei, height; Wei, weight; RVEDVI, right ventricular end diastolic volume index; RVESVI, right ventricular end systolic volume index; RVEF, right ventricular ejection fraction; P-RVOT, proximal right ventricular outflow tract; M-RVOT, mid right ventricular outflow tract; D-RVOT, distal right ventricular outflow tract; L1,proximal end of funnel sleeve to fusion; L2,proximal end of pulmonary trunk to fusion; Ad, average diameter; RVEDVI-P, right ventricular end diastolic volume index postoperation; RVESVI-P, right ventricular end systolic volume index postoperation.

### 3.4 Operative

All 102 patients successfully completed hybrid TPVI. The mean operating time was 239 ± 121 min. The median sternotomy approach was used in 67 cases (65.7%), the subxiphoid approach in 12 cases (11.8%), the left intercostal approach in 12 cases (11.8%), the substernal incision approach in 10 cases (9.8%), and the combined xiphoid and intercostal approach in 1 case (0.9%) (Supplementary Table S3). TPV size selection is summarized in Table 3.

**Table 3:** Distribution of transcatheter pulmonary valve sizes (*N* = 102).

Valve size (mm)	20	22	24	26	28	30	32
*n*	4	3	12	8	12	26	37
%	3.9	2.9	11.8	7.8	11.8	25.5	36.3

A total of 102 patients underwent hybrid TPVI, with concomitant procedures performed in 54 cases (52.9%). The most common concomitant procedures included MPA constriction (24 cases, 23.5%) and PVL plication following TPVI (10 cases, 9.8%). Branch PA interventions were performed in 10 cases (9.8%), including branch arterioplasty for stenosis (*n* = 5, 4.9%), balloon dilatation with PVL plication for septiform stenosis (*n* = 4, 3.9%), and left PA stenting (*n* = 1, 1.0%). Other concomitant procedures included tricuspid valvuloplasty (*n* = 3, 2.9%), of which one was due to intraoperative injury of the tricuspid chordae tendineae; patent ductus arteriosus ligation following PA plication (*n* = 1, 1.0%); double aortic arch correction (*n* = 1, 1.0%); and balloon dilatation for diaphragm-like stenosis of the PT (*n* = 3, 2.9%). Additionally, two patients (1.9%) with intraoperative hemorrhage required concurrent repair of the PA and RV.

Among the 24 patients who underwent MPA constriction, the native RVOT was dilated and/or irregular. Constriction was performed to create a stable, circular landing zone matching the selected valve size. The final landing zone diameter after constriction was 30 mm in all 24 patients, and all 24 patients received a 32 mm mid-section Salus valve. One of these patients also received permanent pacemaker implantation due to pre-existing complete atrioventricular block (III° AVB) diagnosed before the hybrid TPVI procedure. Other representative subsets within this group included one patient with severe PR after prior balloon pulmonary valvuloplasty who underwent transthoracic MPA constriction with TPVI and pulmonary balloon dilatation, and two patients with PR and no prior surgical history who underwent intercostal MPA constriction and TPVI.

There were no intraoperative or immediately postoperative deaths. One patient died on postoperative day 12 during the index hospitalization. This patient was a 30-year-old with a history of transannular patch repair of TOF, whose preoperative RVEDVI was 222.3 mL/m^2^, RVEF was 16.3% and the internal diameter of MPA was 36 mm, the intraoperative median thoracotomy approach with MPA constriction and TPVI was selected, but RV was closely attached to the sternum, and it ruptured during dissection, the operation time was prolonged, the patient developed postoperative low cardiac output, and the death was caused by cardiac arrest 12 days after the operation.

### 3.5 CPB

Among the 102 cases, 20 cases received CPB assistance during the operation. Indications included: TPV dislodgement (*n* = 10), thoracotomy-related hemorrhage (*n* = 2), tricuspid chordae tendineae rupture (*n* = 1), organic TR (*n* = 2), and PA branch non-septate stenosis necessitating arterioplasty (*n* = 5). Among the 10 valve dislodgement cases, 3 occurred after MPA plication without constriction, where the plication alone was insufficient to prevent migration. In the three high-volume centers (≥10 procedures each, total 72 cases), 9 of 10 valve dislodgements occurred; among these, 7 (77.8%) took place within the first half of each center's cumulative experience. The dislodgement rate in these centers was 19.4% (7/36) in the first half vs. 5.6% (2/36) in the second half (*P* = 0.08, Fisher's exact test). A formal time-segmented analysis across all 11 centers was not feasible because procedural volumes varied widely (range 1–37 cases). Comparative analysis revealed no statistically significant differences in baseline characteristics or RVOT dimensions between the CPB-assisted and off-pump groups. However, there was a statistically significant difference in preoperative RV volumes between the CPB group and the off-pump group (*P* < 0.05). It should be noted that there was no statistically significant difference in RV volumes after 1-year follow-up (Supplementary Table S4).

### 3.6 Follow-up

A 1-year follow-up was completed for all patients. Among them, one patient with claustrophobia did not undergo pre- or postoperative CMRI. One patient with unplanned pregnancy and one patient under local COVID-19 lockdown did not undergo postoperative CMRI; these three patients were assessed by transthoracic echocardiography and clinical evaluation. No stent fracture was observed. There were no deaths after discharge during follow-up. Right ventricular volumes (RVEDVI and RVESVI) and QRS duration improved significantly, whereas RVEF remained stable (Table 4).

**Table 4:** Comparison of electrocardiographic and cardiac magnetic resonance imaging parameters before and after intervention.

Parameter	Preoperative	Postoperative	*P* value
QRS duration (ms)	149.74 ± 31.94	137.06 ± 30.74	<0.001
RV voltage (mV)	2.01 ± 1.07	1.72 ± 0.93	<0.001
RVEDVI (mL/m^2^)	147.72 ± 45.69	109.73 ± 32.00	<0.001
RVESVI (mL/m^2^)	84.27 ± 32.67	61.61 ± 24.24	<0.001
RVEF (%)	42.67 ± 9.17	44.02 ± 9.82	0.149

Data are presented as mean ± standard deviation. CMRI-based analysis includes 99 patients with paired preoperative and postoperative CMRI data. Three patients without postoperative CMRI were assessed by echocardiography and clinical follow-up. RV, right ventricular; RVEDVI, right ventricular end-diastolic volume index; RVESVI, right ventricular end-systolic volume index; RVEF, right ventricular ejection fraction.

## 4 Discussion

RVOT morphology is a primary factor influencing the choice of TPVI approach. Percutaneous TPVI is suitable for most patients, except those with type I RVOT morphology or RVOT dilation (18). For patients with a dilated RVOT requiring downsizing, the selection between a hybrid approach and CPB assistance primarily depends on the presence of concomitant intracardiac anomalies requiring simultaneous intervention and the risk of TPV dislodgement or embolization. TPV sizing is predominantly determined by the diameter at the annular level of the intended anchoring zone. Therefore, RVOT morphology and diameter are all crucial intraoperative considerations.

The choice of surgical approach was primarily based on RVOT morphology, as previously mentioned. In the early phase of program implementation across most centers, the median sternotomy approach was more frequently selected due to concerns regarding intraoperative bleeding during re-sternotomy and the risk of valve displacement during deployment. However, with accumulated experience, it was observed that reoperative median sternotomy was associated with more severe adhesions, whereas the intercostal approach resulted in less mediastinal tissue adhesion, leading to a smaller surgical field and shorter duration of postoperative drainage. Furthermore, in cases with significant RV enlargement, the median sternotomy approach often resulted in a challenging angle between the delivery sheath and the MPA, increasing the difficulty of achieving optimal coaxial valve deployment. In contrast, the left fourth intercostal approach provided a more favorable sheath-to-MPA trajectory, facilitating easier and more coaxial valve implantation. For patients with a regular-shaped RVOT and an inner diameter ≤30 mm, a subxiphoid or left fourth intercostal incision was adopted. For those with an irregular RVOT morphology presenting a higher risk of PVL or device displacement, a strategy involving MPA plication or constriction through a left second intercostal incision combined with either a left fourth intercostal incision, a lower partial sternotomy, or a full median sternotomy was employed. Patients with concomitant malformations requiring repair under CPB or those with great vessel anomalies underwent median sternotomy. Median sternotomy was reserved for cases requiring concurrent repair of associated intracardiac defects or other necessary cardiac interventions.

In this study, CPB was utilized in 20 cases. The need for CPB was determined intraoperatively in 13 cases for managing unforeseen events (valve dislodgement, hemorrhage, chordae injury). In the three high-volume centers (≥10 procedures each, total 72 cases), 9 of 10 valve dislodgements occurred; among these, 7 (77.8%) took place within the first half of each center's cumulative experience. The high dislodgement rate reflects the challenging cohort with large, irregular native RVOTs and a learning curve. Technical modifications (CTA-based sizing, liberal MPA constriction/plication and fixation sutures) likely reduced later events. All dislodgements were managed intraoperatively without long-term sequelae. For the remaining 7 cases, CPB was planned preoperatively for concurrent tricuspid valve repair (*n* = 2) or extensive branch arterioplasty (*n* = 5). In these planned cases, we still chose hybrid TPVI over surgical PVR to utilize the TPV stent to remodel the irregular RVOT (creating a favorable anatomy for future valve-in-valve procedures) and to avoid a suture line on a potentially friable RVOT, potentially reducing CPB time. Based on preoperative data comparison, these patients had worse cardiac function; however, there was no statistically significant difference in the recovery of RV volumes postoperatively. These findings suggest that CPB support may not preclude postoperative RV functional recovery. However, given the small sample size (*n* = 20), significant baseline differences in RVEDVI and RVESVI, and the lack of adjustment for confounders, no definitive conclusion can be drawn. Larger studies with propensity matching are needed to determine whether CPB assistance independently affects postoperative RV recovery. Cases involving valve dislodgement and tricuspid chordae tendineae rupture were attributed to the learning curve of the operators.

CAC represents one of the most critical complications in percutaneous TPVI, particularly for patients with failed RV-to-PA conduits, in which the conduit often lies in close proximity to the coronary arteries and directly behind the sternum (19). To mitigate this risk, strategies such as intraoperative balloon inflation with simultaneous coronary angiography (balloon testing) (20), as well as pre-procedural assessment of the coronary-RVOT and sternum-RVOT distances via CMRI (21), have been developed and have significantly reduced the incidence of CAC in TPVI. It should be noted that patients with severe pulmonary hypertension and PA dilation are also at risk of CAC, which has led researchers to propose an anatomical classification system for the relationship between the coronary arteries and the PA (22, 23). Importantly, the risk profile differs substantially between patients with failed conduits and those with dilated native RVOTs after prior repair. In our cohort of patients with native RVOT dilation, the anatomical space between the RVOT and coronary arteries is typically sufficient. Additionally, intraoperative MPA constriction reduces RVOT volume, and valve size is meticulously matched to the inner diameter of the landing zone, further minimizing CAC risk. Nevertheless, rare cases of coronary compression after self-expanding valve implantation in native RVOTs have been reported, primarily attributed to oversizing (24, 25). During the early phase of our clinical experience, we routinely performed balloon inflation testing to preemptively exclude CAC. As our experience matured, preoperative risk assessment for patients without anomalous aortic origin of coronary arteries primarily relied on CTA-based measurements of the distance between the coronary arteries and the RVOT. Balloon testing was reserved for cases deemed at higher risk based on imaging. Ultimately, in this series of 102 patients, no CAC events occurred.

According to relevant studies, the incidence of infective endocarditis is approximately 1.6%–2.7%, primarily observed in patients with a history of infective endocarditis, DiGeorge syndrome, or increased residual gradient following balloon-expandable TPVI (26, 27). One suspected case in our cohort had persistent low-grade fever for 2 weeks but was managed at a local primary care facility during the COVID-19 pandemic, where echocardiography and blood cultures were unavailable. Empirical second-generation cephalosporin resolved the fever; antibiotics were continued for 4 weeks. The diagnosis remained unconfirmed, and no other cases occurred.

Ventricular arrhythmias, particularly premature ventricular contractions, often result from mechanical irritation of the infundibular myocardium by the valve stent (28, 29). These are more frequently observed in cases with an excessively short RVOT or an excessively low valve implantation position. Owing to its shorter stent length compared to other self-expanding valves, the Salus TPV is associated with a lower incidence of ventricular arrhythmias.

TPV stent fracture primarily occurs due to mismatch between the valve and the inner diameter of the RVOT or excessively low implantation positioning reaching the infundibulum (3–5, 30, 31). It is more frequently observed with balloon-expandable valves and percutaneous TPVI. In our cohort, no stent fracture was observed during the 1-year follow-up period. The absence of stent fractures may be attributed to a sizing strategy that integrates comprehensive assessment of RVOT morphology—including the mid-MPA circumference-derived diameter, overall length, degree of irregularity, and morphological type—with a safety margin ≥2 mm relative to the landing zone diameter. However, longer follow-up is needed to confirm this observation.

Postoperative PA branch stenosis is a common complication following NRVOTR and can also exacerbate PR. It is noteworthy that few studies in the field of TPVI have addressed the concurrent management of this complication. In our cohort, five cases with diffuse branch stenosis underwent concomitant pulmonary arterioplasty under CPB support. Additionally, four cases with diaphragm-like stenosis at the origin of the branch pulmonary arteries were treated with balloon angioplasty. Postoperative echocardiographic follow-up showed no residual branch stenosis in these patients.

TR is another common long-term complication of severe PR, often secondary in nature, though some cases result from iatrogenic injury during initial surgery or TPVI. In our series, two cases with organic moderate-to-severe TR and one case with severe functional TR in a low-body-weight patient underwent concurrent tricuspid valve repair under CPB support. Among three additional patients with secondary moderate-to-severe TR, two—with preserved RV function and a high likelihood of postoperative recovery—received only TPVI without tricuspid intervention. The third patient, who presented with massive pleural effusion, ascites, and congestive hepatopathy, was deemed at high surgical risk under CPB and therefore also underwent TPVI alone. At 1-year follow-up, one of the two patients with good preoperative RV function showed mild TR, while the other (residing in the high-altitude region of Tibet) had moderate-to-severe TR. The high-risk patient showed moderate-to-severe TR at 1-year follow-up but reported significant improvement in exercise tolerance and absence of facial or limb edema.

## 5 Study limitations

This study has several limitations. The retrospective, multicenter design introduces potential selection bias and variability in procedural technique and postoperative management across participating institutions. Because of the exploratory, hypothesis-generating design and the small sample sizes within subgroups, no adjustment for multiple comparisons was applied, and no *a priori* power calculation was performed. Subgroup analyses were *post hoc*; Bonferroni-type correction would have increased the risk of Type II error. Therefore, the absence of statistically significant differences across RVOT morphology types should not be interpreted as evidence of no difference, and all subgroup results should be regarded as descriptive and hypothesis-generating.

Imaging and echocardiographic data were assessed locally rather than in a central core laboratory, and inter-observer variability was not systematically assessed, which may introduce measurement variability across centers.

The CPB subgroup was small and had worse preoperative right ventricular volumes; therefore, comparisons between CPB and off-pump groups are exploratory and may be confounded.

Follow-up remains relatively short, with a median of 1 year. Long-term data are essential to evaluate the durability of the Salus TPV, long-term hemodynamic performance, and late complications such as endocarditis or stent fracture. Patients requiring reintervention after NRVOTR are generally young and face the prospect of multiple valve-related interventions; as seen with mitral and aortic valves, younger age is a risk factor for accelerated valve deterioration. Long-term follow-up after TPVI is therefore critical.

TR was not a pre-specified endpoint. One-year follow-up across centers focused primarily on pulmonary valve function, and TR grade was documented only as routine clinical data without centralized assessment. Thus, a complete summary of TR grade changes was not available for the whole cohort, and detailed data are limited to the cases described in the Discussion.

Future prospective studies with longer follow-up and standardized core laboratory assessment are needed to validate these findings.

## 6 Conclusion

This multicenter study demonstrates that hybrid TPVI using the Salus self-expanding valve is safe and effective for patients with moderate-to-severe PR and complex native RVOTs who are not candidates for percutaneous intervention.

In 102 patients, procedural success was 100% with no intraoperative mortality. At 1-year follow-up, RV volumes significantly improved and QRS duration decreased, with stable RVEF; no coronary compression, valve embolization, or stent fracture occurred. Adverse events were rare, with one suspected endocarditis and one in-hospital death on postoperative day 12 in a high-risk patient.

Individualized preoperative planning based on RVOT morphology is essential for optimal outcomes. Longer-term follow-up is needed to assess valve durability.

## Data Availability

The original contributions presented in the study are included in the article/Supplementary Material, further inquiries can be directed to the corresponding authors.
