# Supplementary material for: Multicenter experience of hybrid transcatheter pulmonary valve implantation in patients with a large right ventricular outflow tract
Source: Front Cardiovasc Med. 2026 Oct 20;13:1815136. doi: 10.3389/fcvm.2026.1815136 (PMC13727611; doi:10.3389/fcvm.2026.1815136)
Supplement: Supplementary file 1 [file Table1.docx]

Supplementary Material

Supplementary Table 1 Baseline Characteristics of the Study Cohort (N=102)

| **Variables** | **Value (mean ± SD) / N (%)** |
| --- | --- |
| **Demographics** |  |
| Age (years) | 22.37 ± 11.15 |
| Gender (Male: Female) | 58 (56.9%): 44 (43.1%) |
| Height (cm) | 161.59 ± 12.45 |
| Weight (kg) | 55.00 ± 16.35 |
| Years since last operation | 15.22 ± 5.14 |
| **Primary Anatomic Diagnosis** |  |
| Tetralogy of Fallot | 75 (73.5%) |
| Pulmonary stenosis | 10 (9.8%) |
| Pulmonary atresia with VSD | 3 (2.9%) |
| Other diagnoses * | 14 (13.7%) |
| **Type of previous cardiac operation** (N=99) |  |
| Transannular patch repair | 71 (71.7%) |
| Pulmonary valve plasty | 22 (22.2%) |
| Other procedures † | 6 (6.1%) |

*Other diagnoses include: TOF/APV (absent pulmonary valve syndrome) (2.0%), TOF/AVSD (atrioventricular septal defect) (1.0%), TOF/ASD (atrial septal defect) (2.0%), PA/IVS (pulmonary atresia with intact ventricular septum) (2.0%), DORV (double outlet right ventricle) (2.0%), isolated PR (pulmonary regurgitation) (2.0%), MR/TR/VSD (mitral regurgitation/tricuspid regurgitation/ventricular septal defect) (1.0%), Ruptured SVA/PAA (sinus of Valsalva aneurysm/pulmonary artery aneurysm) (1.0%), TOF/DAA (double aortic arch) (1.0%).

†Other procedures include: balloon valvuloplasty, Rastelli procedure, Barbero-Marcial procedure, shunt operations, etc.; SD, standard deviation.

Supplementary Table 2 Comparison of Pre- and Post-operative Parameters by RVOT Morphology Type

| **Parameter** | **Type I (n=38)** | **Type II (n=31)** | **Type III (n=16)** | **Type IV (n=6)** | **Type V (n=11)** | ***P* Value** |
| --- | --- | --- | --- | --- | --- | --- |
| **RVOT dimensions (mm)** |  |  |  |  |  |  |
| Infundibulum diameter | 39.05 ± 8.33 | 33.81 ± 7.81 | 34.02 ± 7.45 | 28.72 ± 3.59 | 35.83 ± 11.75 | 0.016 |
| Mid-RVOT diameter | 32.15 ± 6.56 | 31.34 ± 6.19 | 31.67 ± 7.12 | 33.48 ± 6.07 | 26.09 ± 8.22 | 0.127 |
| Distal-RVOT diameter | 28.52 ± 6.25 | 30.91 ± 6.24 | 36.46 ± 7.27 | 27.70 ± 5.93 | 34.40 ± 11.13 | 0.002 |
| **Pre-operative function** |  |  |  |  |  |  |
| RVEDVI (mL/m²) | 149.82 ± 48.88 | 145.76 ± 42.47 | 134.90 ± 27.19 | 154.55 ± 38.22 | 162.28 ± 68.76 | 0.644 |
| RVEF (%) | 41.13 ± 10.25 | 43.29 ± 8.31 | 45.74 ± 8.16 | 43.82 ± 13.13 | 41.05 ± 5.76 | 0.498 |
| **Post-operative function** |  |  |  |  |  |  |
| RVEDVI (mL/m²) | 105.43 ± 31.06 | 113.28 ± 33.70 | 109.12 ± 23.49 | 107.88 ± 18.75 | 117.19 ± 48.26 | 0.809 |
| RVEF (%) | 45.10 ± 8.55 | 43.29 ± 10.21 | 47.38 ± 8.55 | 38.95 ± 12.00 | 39.79 ± 12.59 | 0.202 |

Data presented as mean ± SD; RVOT: right ventricular outflow tract; RVEDVI: right ventricular end diastolic volume index; RVEF: right ventricular ejection fraction.

Supplementary Table 3 Procedural Details and Concomitant Procedures

| Variable | N (%) |
| --- | --- |
| **Surgical approach** |  |
| Median sternotomy | 67 (65.7%) |
| Subxiphoid | 12 (11.8%) |
| Left intercostal | 12 (11.8%) |
| Substernal incision | 10 (9.8%) |
| Combined xiphoid/intercostal | 1 (1.0%) |
| Operation time (minutes), mean ± SD | 239 ± 121 |
| **Concomitant procedures** |  |
| Isolated TPVI | 48 (47.1%) |
| TPVI + pulmonary constriction | 24 (23.5%) |
| TPVI + PVL plication | 10 (9.8%) |
| TPVI + branch arterioplasty | 5 (4.9%) |
| TPVI + balloon dilation + PVL plication | 4 (3.9%) |
| TPVI + other * | 11 (10.8%) |
| Cardiopulmonary bypass support | 20 (19.6%) |

Other procedures include left pulmonary artery stenting, tricuspid valvuloplasty, patent ductus arteriosus ligation, etc., as detailed in the manuscript.

TPVI, Transcatheter Pulmonary Valve Implantation; PVL, Paravalvular Leakage; SD, standard deviation.

Supplementary Table 4 Subgroup Analysis: Patients Requiring Cardiopulmonary Bypass Support

| **Parameter** | **Off-Pump (n=82)** | **CPB-Support (n=20)** | ***P* Value** |
| --- | --- | --- | --- |
| **Pre-operative** |  |  |  |
| RVEDVI (mL/m²) | 141.14 ± 40.72 | 176.14 ± 55.55 | 0.002 |
| RVESVI (mL/m²) | 80.83 ± 29.72 | 99.14 ± 40.84 | 0.027 |
| RVEF (%) | 42.31 ± 9.68 | 44.23 ± 6.47 | 0.415 |
| **Post-operative metrics** |  |  |  |
| RVEDVI (mL/m²) | 107.42 ± 29.32 | 119.71 ± 41.15 | 0.132 |
| RVESVI (mL/m²) | 61.06 ± 23.76 | 63.98 ± 26.76 | 0.639 |
| RVEF (%) | 43.96 ± 10.40 | 44.26 ± 7.05 | 0.905 |
| **Post-operative improvement (Δ)** |  |  |  |
| ΔRVEDVI (mL/m²) | 33.72 ± 31.98 | 56.43 ± 51.74 | 0.016 |
| ΔRVESVI (mL/m²) | 19.76 ± 25.31 | 35.16 ± 38.10 | 0.034 |
| ΔRVEF (%) | -1.65 ± 9.81 | -0.03 ± 6.49 | 0.497 |

Data presented as mean ± SD; RVEDVI: Right Ventricular End-Diastolic Volume Index; RVESVI: Right Ventricular End-Systolic Volume Index; RVEF: Right Ventricular Ejection Fraction.

Supplementary Table 5 Number of hybrid TPVI procedures performed at each participating center

| Center | Procedures (n) |
| --- | --- |
| Beijing Anzhen Hospital | 24 |
| Guangdong Provincial People's Hospital | 37 |
| Fuwai Central China Cardiovascular Hospital | 10 |
| West China Hospital, Sichuan University | 5 |
| Children's Hospital of Fudan University | 8 |
| Zhongnan Hospital of Wuhan University | 5 |
| Beijing Huaxin Hospital | 5 |
| Kunming Yan'an Hospital | 3 |
| Union Hospital, Tongji Medical College, HUST | 2 |
| The First Affiliated Hospital of Guangxi Medical University | 2 |
| The First Affiliated Hospital of Zhengzhou University | 1 |
| **Total** | **102** |
